# An alternative vaccine target for bovine Anaplasmosis based on enolase, a moonlighting protein

**DOI:** 10.3389/fvets.2023.1225873

**Published:** 2023-09-22

**Authors:** Rosa Estela Quiroz-Castañeda, Hugo Aguilar-Díaz, Itzel Amaro-Estrada

**Affiliations:** Centro Nacional de Investigación Disciplinaria en Salud Animal e Inocuidad, INIFAP. Carretera Federal Cuernavaca-Cuautla, Col. Progreso, Jiutepec, Morelos, Mexico

**Keywords:** multifunctional proteins, enolase, erythrocytes, ticks, extracellular matrix, veterinary diseases

## Abstract

The discovery of new targets for preventing bovine anaplasmosis has moved away from focusing on proteins that have already been extensively studied in *Anaplasma marginale*, including the Major Surface Proteins, Outer Membrane Proteins, and Type IV Secretion System proteins. An alternative is moonlighting or multifunctional proteins, capable of performing various biological functions within various cellular compartments. There are several reports on the role of moonlighting proteins as virulence factors in various microorganisms. Moreover, it is known that about 25% of all moonlighting is involved in the virulence of pathogens. In this work, for the first time, we present the identification of three enolase proteins (AmEno01, AmEno15, and AmEno31) in the genome of Mexican strains of *A. marginale*. Using bioinformatics tools, we predicted the catalytic domains, enolase signature, and amino acids binding magnesium ion of the catalytic domain and performed a phylogenetic reconstruction. In addition, by molecular docking analysis, we found that AmEno01 would bind to erythrocyte proteins spectrin, ankyrin, and stomatin. This adhesion function has been reported for enolases from other pathogens. It is considered a promising target since blocking this function would impede the fundamental adhesion process that facilitates the infection of erythrocytes. Additionally, molecular docking predicts that AmEno01 could bind to extracellular matrix protein fibronectin, which would be significant if we consider that some proteins with fibronectin domains are localized in tick gut cells and used as an adhesion strategy to gather bacteria before traveling to salivary glands. Derived from the molecular docking analysis of AmEno01, we hypothesized that enolases could be proteins driven by the pathogen and redirected at the expense of the pathogen’s needs.

## 1. Introduction

The performance of more than one function (moonlighting) by a single protein has been recognized as a common phenomenon with significant implications in metabolic processes and other functions in bacteria, plants, yeasts, fungi, parasites, and vertebrates (1–8). Moonlighting proteins were described in the late 1980s as structural proteins in the lens of the eye (crystallins) with a second and even a third function (9–11). Most of the functions of moonlighting proteins are related to physiologically relevant biochemical or biophysical functions (12–14). The repertoire functions of moonlighting proteins reported in the last years include their participation as enzymes of the TCA cycle (aconitase, *Homo sapiens, Saccharomyces cerevisiae, Mycobacterium tuberculosis*) (15, 16); glucose metabolism (aldolase, *Arabidopsis thaliana*, hexokinase; *Plasmodium vivax*) (17, 18); chaperones (GroEL, *S. cerevisiae*, HSP60; *Enterobacter aerogenes*) anti-oxidant proteins (thioredoxin, *Escherichia coli*) (19); virulence-associated functions (elongation factor Tu and enolase, *Mycoplasma pneumoniae*, *Pseudomonas aeruginosa, Streptococcus pneumoniae, Plasmodium* spp.) (20, 21); among other roles in different organisms that have been widely reported (6, 22–24) In the last years, due to sequencing technologies and metagenomics advances, more moonlighting proteins and their functions are being discovered in diverse organisms (25).

Currently, two large groups have been proposed to classify moonlighting proteins: 1) “trigger enzymes” and 2) intracellular/secreted moonlighting proteins (4, 26). The first subset comprises enzymes that regulate transcription or translation by directly binding to DNA or RNA or by binding to other proteinaceous translation or transcription factors (27). The second subset includes the most extensive known moonlighting proteins, with activities as housekeeping enzymes, chaperones, translation factors, adhesion, DNA-binding proteins, and many others that are secreted and either reside attached to the cell surface, acting as receptors for soluble proteins or small molecules, or function in the fluid phase, often for intracellular signaling (4). In this regard, as moonlighting proteins perform their canonical and moonlighting functions in separate cell compartments (cytoplasm and the cell surface), this dual cellular localization of the protein strongly suggests a multifunctional activity (28).

The presence of moonlighting proteins is relevant in bacteria, and their study focuses on elucidating their alternative functions since they are present in both pathogenic and commensal bacteria (23).

In this regard, *Anaplasma marginale* is a Gram-negative intracellular pathogen known as the causal agent of bovine Anaplasmosis, an infectious, non-contagious disease characterized by progressive hemolytic anemia, abortions, loss of condition, milk production, and even death (6, 29, 30). Up to now, seven genomes (~1.2 Mbp) of Mexican strains of *A. marginale* have been reported and annotated (31–33). Due to their reduced genome size, moonlighting proteins in this pathogen could be a strategy to efficiently maximize their proteins’ use (31, 34).

Currently, our interest focuses on moonlighting proteins of this vector-borne pathogen since they could participate in its pathogenicity or evasion of the host immune system, as it has been reported for many pathogens, which employ moonlighting/multitasking proteins as virulence factors to interfere with multiple cellular processes, in different compartments at different times during infection, augmenting their virulence (6). Thus, we performed a deep and sharp genomic analysis that allowed us to identify potential moonlighting proteins in *A. marginale*, including enolase (AmEno), which have not been reported before in this pathogen. Enolase (2-phospho-D-glycerate hydrolase, EC 4.2.1.11) is an intensely studied moonlighting protein that converts 2-phosphoglycerate to phosphoenolpyruvate in glycolysis. Besides participating in this metabolic pathway, the enolase facilitates binding to host cells, as reported in *Anaplasma phagocytophilum*, in which enolase binds to the host plasminogen. In 2018, Gao et al. (35) demonstrated that recombinant enolase from *A. phagocytophilum* can bind and activate plasminogen and promote conversion to plasmin, thus being crucial to pathogen infection. In addition, this enolase was considered a potential target to control anaplasmosis infection. In *Borrelia burgdorferi*, the surface-expressed enolase plays an essential role during pathogen invasion by binding mammalian plasminogen (36, 37). Recently, Xie et al., (38) confirmed that *Mycoplasma hyopneumoniae* enolase is localized on its surface and is capable of adhesion to swine tracheal epithelial cells.

In this work, we performed bioinformatic analysis, three-dimensional (3D) modeling, and docking of the *A. marginale* enolase, AmEno. This study aimed to identify *in silico* their potential to interact with different proteins from the extracellular matrix (ECM), erythrocyte membrane (EM), and the zymogen plasminogen that circulates in the mammals’ blood. In addition, it could guide the development of a rational and sharp strategy to understand the interaction and functions of enolase and some ligands, which are essential for the success of the pathogen establishment.

## 2. Materials and methods

### 2.1. Identification of moonlighting enolases in *Anaplasma marginale* genomes

All seven *A. marginale* draft genomes reported (MEX-01-001-01, MEX-14-010-01, MEX-15-099-01, MEX-17-017-01, MEX-30-184-02, MEX-30-193-01, and MEX-31-096-01) had been previously annotated automatically using the RAST (version 2.0) server (39). Derived from this annotation, we identified one enolase gene in each of the seven genomes. Besides, sequences of enolases from different organisms were retrieved from a Blastp search at NCBI. Additionally, a search in AlphaFold Protein Structure Database (40) and MoonProt 2.0 (41) allowed a comparison of *A. marginale* enolase with those reported as pathogen virulence proteins. The selected sequences were used in the phylogenetic reconstruction.

### 2.2. Phylogenetic reconstruction

Enolase sequences were selected from the Domains Eukarya and Bacteria (Table S1). All multiple alignments were performed with Clustal Omega (42) and visualized with Jalview (43). A neighbor-joining method was used to reconstruct a phylogeny using Mega 11 software (44) with a Poisson substitution model and a bootstrap value of 1,000 replicates.

### 2.3. Bioinformatics analyses of *Anaplasma marginale* enolases

The magnesium (Mg^2+^) binding sites, which are essential for the catalytic activity of the enolase and the conserved domains, were predicted in the Conserved Domains database (CDD-NCBI) (44) and ScanProsite (45). Transmembrane regions were predicted with DeepTMHMM (46). The subcellular localization of the proteins was predicted in PSORTb 3.0 (46), and the secondary structure and function were predicted in PSIPRED Workbench (47). The topology of the proteins was predicted in CATH (48).

### 2.4. Three-dimensional (3D) modeling

SwissModel is a protein structure homology-modeling server widely used to predict the 3D structure of proteins (49). We used this server to predict the 3D structures of the *A. marginale* enolases from strains MEX-01-001-01 (AmEno01), MEX-15-099-01 (AmEno15), and MEX-31-096-01 (AmEno31). The rest of the enolases from strains MEX-30-193-01, MEX-30-184-02, MEX-14-010-01, and MEX-17-017-01 are essentially identical to MEX-01-001-01; consequently, these structures were not modeled.

Homology modeling is currently an accurate method to generate reliable 3D protein structure models, using experimental protein structures from PDB (“templates”) to build models for evolutionary-related proteins (“targets”). All generated models in SwissModel are based on the GMQE (Global Model Quality Estimate) and QMEAN model quality.

### 2.5. Molecular docking and interaction analysis

The docking of the modeled AmEno01 and five possible ligands was performed in ClusPro (50) to analyze their binding affinity. The PDB ID numbers of the ligands are plasminogen (4DUR), Fibronectin (3M7P), Spectrin (3LBX), Ankyrin (4RLV), and Stomatin (4FVF).

ClusPro is a server that uses a fast Fourier transform (FFT) method called Piper, where one of the proteins is placed at the origin of the coordinate system on a fixed grid, the second protein is placed on a movable grid, and the interaction energy is written as a sum of a few correlation functions. The algorithm rotates the ligand with 70,000 rotations. The 1,000 rotations/translation combinations out of the 70,000 rotations with the lowest score are chosen, and these 1,000 ligand positions are clustered with a 9 Å C-alpha RMSD radius (51). In ClusPro, we generated docking models using AmEno01 and the five potential ligands. For each molecular docking, ten models were generated that were downloaded in PDB format and visualized in UCSF ChimeraX (52). The model with the highest score of the ten docking models was selected to visualize the contact surface model in HDOCK (53).

The analysis of the interactions between amino acids of the five AmEno01-ligand complexes and the visualization were performed in PDBsum (54). For this, we first used the option PDBsum Generate to upload each docking model and generate a PDB code. Once we retrieved the PDB code of the five docking models, we used it as an entry in PDBsum to analyze the AmEno01-ligand interactions.

## 3. Results

### 3.1. Identification of moonlighting enolases in Mexican *Anaplasma marginale* genomes

**Figure 1:**
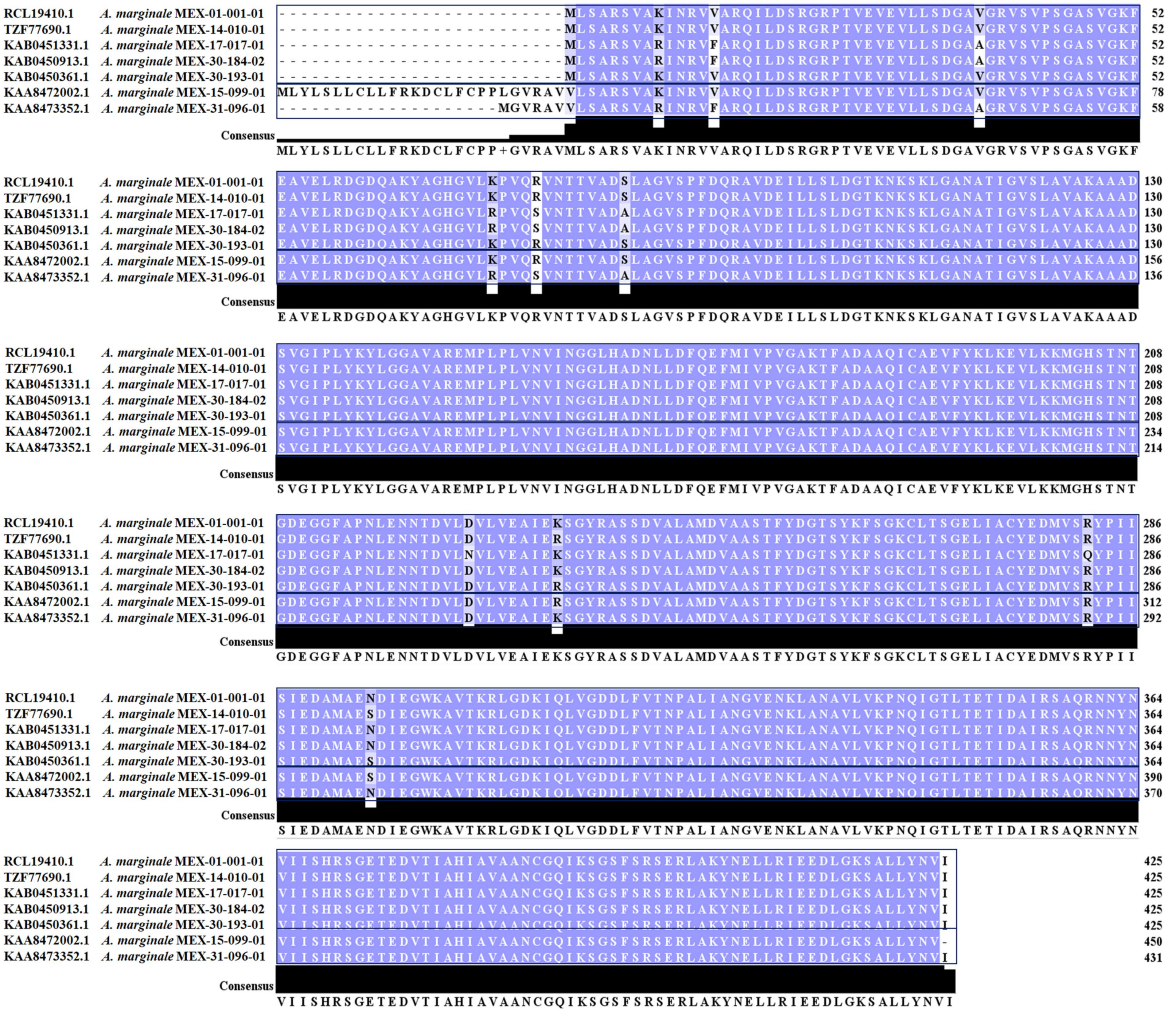
Multiple alignments of *A. marginale* Mexican strains enolases using Clustal Omega. Enolases are classified into three Groups. Group 1 comprises strains MEX-01-001-01, MEX-14-010-01, MEX-17-017-01, MEX-30-184-02, and MEX-30-193-01. These sequences share the same length of 425 aa. Group 2 comprises strain MEX-15-099-01 with 450 aa. Notice the 26 additional amino acids in the N-terminal end. Group 3 comprises MEX-31-096-01 with 431 aa, including six additional amino acids in the N-terminal end. The percentage of identity ranges from 97.41–99.76%.

We identified one enolase per genome in the seven *A. marginale* Mexican strains. The enolases of strains MEX-30-184-02 (GenBank KAB0450913.1), MEX-17-017-01 (KAB0451331.1), MEX-30-193-01 (KAB0450361.1), MEX-14-010-01 (TZF77690.1), and MEX-01-001-01 (RCL19410.1) had 425 amino acids of length. The enolase of strain MEX-15-099-01 (KAA8472002.1) had 450 amino acids, including 26 additional amino acids (MLYLSLLCLLFRKDCLFCPPLGVRAV) in the N-terminal end, and finally, the enolase of strain MEX-31-096-01 (KAA8473352.1) had 431 amino acids, considering six additional amino acids (MGVRAV) in the N-terminal end. Only eleven differences in amino acid sequences were observed in the seven enolase sequences (Figure 1). We performed bioinformatics predictions to all the enolases of the three Groups; however, the modeling and docking analyses we present here were performed only with the strain MEX-01-001-01, AmEno01 sequence. This model was representative of the three *A. marginale* enolases. Nevertheless, to confirm that 3D modeling and molecular docking of the enolases of Groups 2 and 3 did not vary substantially due to the differences at the sequence level, we also performed a docking of these proteins with the ligands. However, we found no significant variation at the structure or interaction level (data not shown).

### 3.2. Phylogenetic reconstruction

**Figure 2:**
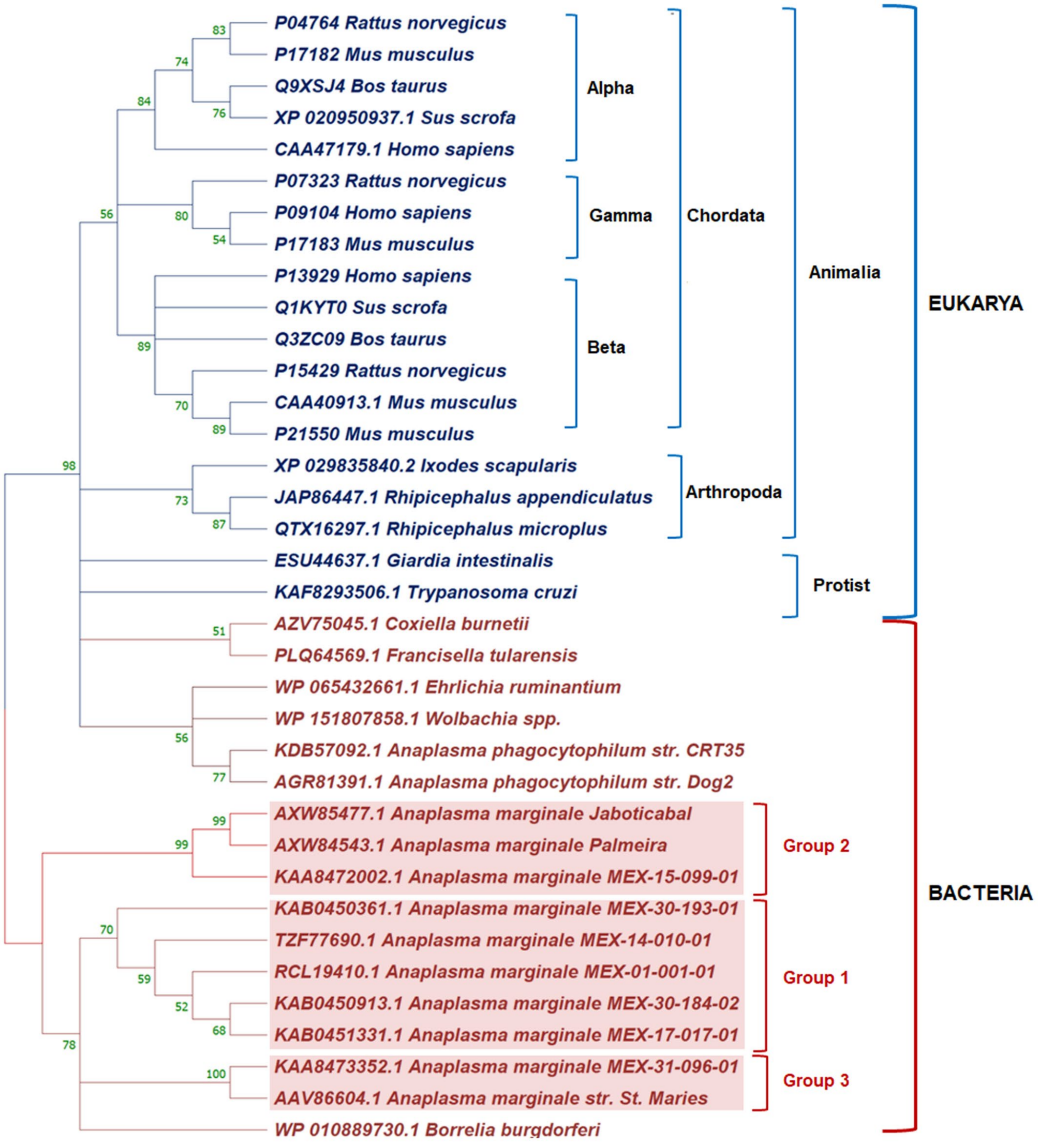
Phylogenetic reconstruction of enolases from Domains Eukarya and Bacteria in Mega 11. In Eukarya, Chordata’ enolases are organized into alpha, beta, and gamma groups. Tick enolases form a clade separated from Animalia and Protist. In Bacteria, the Mexican strains of *A. marginale* grouped with Brazilian strains and North American strains (red boxes).

We reconstructed a phylogenetic tree to determine the phylogenetic relationship of AmEno01 and other enolases reported. It is known that enolases are well-defined in alpha, beta, and gamma groups in mammals. In the phylogenetic reconstruction, we found this classification in Chordata. The enolases of ticks *Rhipicephalus* spp. and *Ixodes scapularis* were clustered in a clade belonging to Arthropoda, and those from Protist organisms were separated from Animalia. In Bacteria, enolases MEX-01-001-01 (AmEno01), MEX-14-010-01 (AmEno14), MEX-17-017-01 (AmEno17), MEX-30-184-02 [(AmEno30-02), and MEX-30-193-01 (AmEno30-01)] were clustered in a unique clade that we named Group 1. The enolase MEX-15-099-01 (AmEno15) and Brazilian strains Jaboticabal and Palmeira clustered in Group 2. Finally, the enolase MEX-31-096-01 (AmEno31) was clustered with the reference strain *A. marginale* St. Maries in Group 3 (Figure 2). Additionally, the sequence identity of Mexican strains ranged from 97.65 to 99.53% in Group 1; the sequence identity between AmEno15 and Brazilian strains was 100%; and the identity between AmEno31 and the reference strain St. Maries was 98.38%.

### 3.3. Bioinformatics analyses of *Anaplasma marginale* enolases

**Figure 3:**
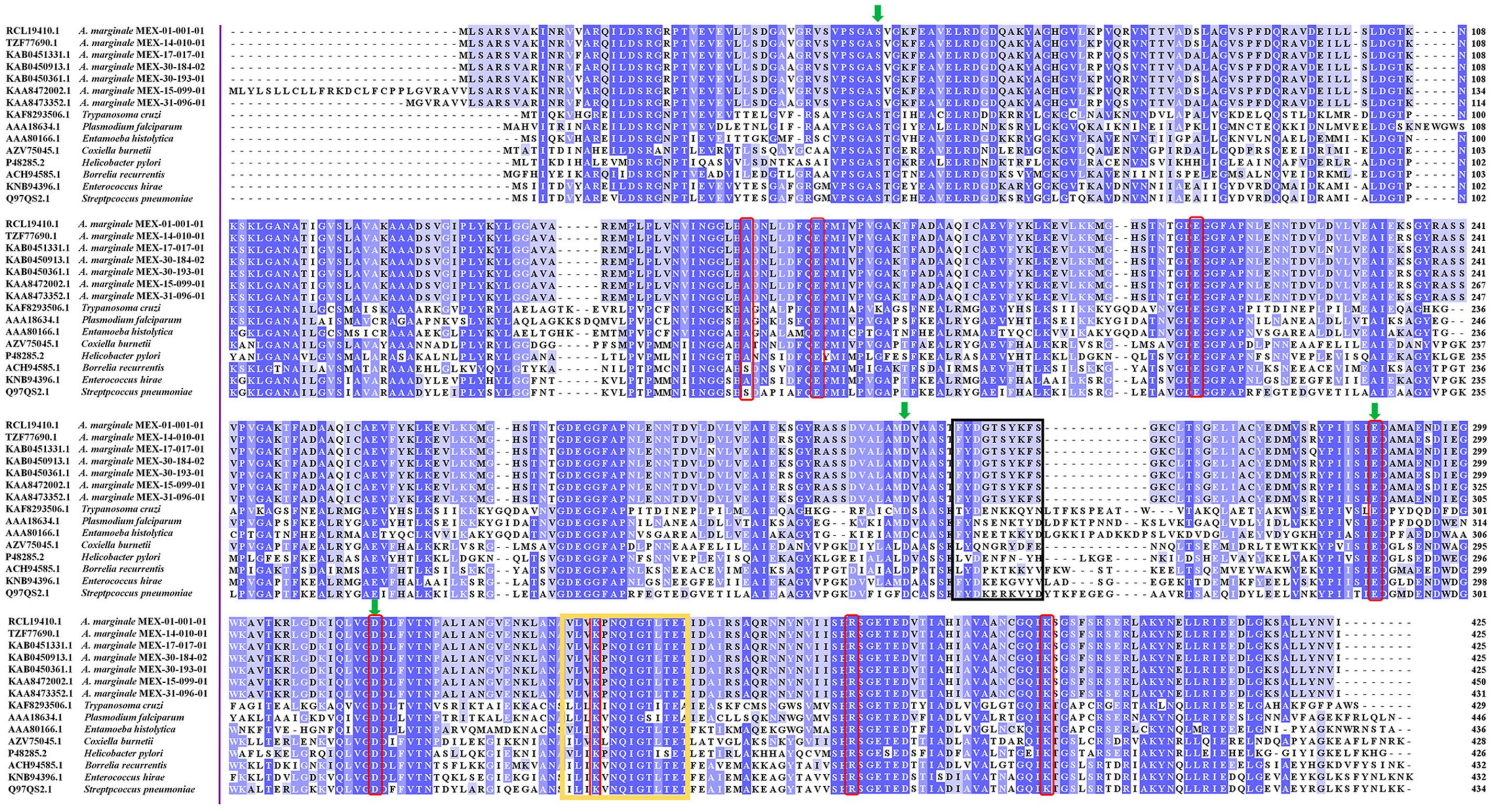
Enolases of parasites and bacteria aligned with Clustal Omega. The plasminogen binding sequence is shown in the black box. The sequence FYDGTSYKFS in *A. marginale* strains varies by 70% from the reported in *S. pneumoniae*, which binds to plasminogen by the sequence FYDKERKVYD. The enolase signature in *A. marginale* strains has seven amino acid changes compared to the sequences shown in the yellow box. The amino acids binding magnesium atoms are shown in green arrows and the amino acids of the catalytic site are shown in red boxes.

The analysis in the CDD database showed four Mg^2+^ binding sites identified in AmEno01, AmEno14, AmEno17, AmEno30-02, AmEno30-01, AmEno15, and AmEno31, which were S, D, E, and D, varying in position along the sequences (Table 1). These residues are significant because of their role in the enolase catalytic activity. The enolase signature was identified in the analysis of ScanProsite. Additionally, DeepTMHMM predicted a localization inside the cell for all enolases since no transmembrane regions were identified, and no signal peptide was predicted with PSORT. Beta strands, alpha helixes, and coils were also identified (Supplementary Figure S1). To identify and compare the sequences of enolase signature, the amino acids of the catalytic site, and the loops of the active site, we contrasted enolases AmEno01, AmEno14, AmEno17, AmEno30-02, AmEno30-01, AmEno15, and AmEno31with information previously reported for *bona fide* enolases of *H. sapiens, T. cruzi, S. pneumoniae, A. phagocytophilum* and, *R. microplus* (Table 1). We found that the amino acids with a significant role in the catalytic site of the enolases were essentially H, E, E, E, D, K, R, S, and K, which varied in the sequence position. Additionally, we found variants of the enolase signature and plasminogen-binding site in Mexican strains (Table 1 and Figure 3).

**Table 1:** Features of some enolases reported for animals, protists, and bacteria, including *A. marginale* enolases.

Species (accession number and length)	Enolase signature	Mg^2+^ binding sites	aa catalytic sites	Loops of active site	Plasminogen binding sequence	Ref.
*H. sapiens* (Alpha enolase, P06733; 434 bp)	^340^LLLKVNQIGSVTES^353^	S^40^ D^245^ E^293^ D^318^	H^158^ E^167^ E^210^ E^293^ D^318^ K^343^ R^372^ S^373^ K^394^	^37^SGASTGIY^44^ ^157^SHAGNKLA^164^ ^248^ASEFFRSGKYDLD FKSPDDPSRYI^271^	^251^FFRSGKY^257^	(55)
*R. microplus* **(QTX16297.1; 434 bp)**	^341^LLLKVNQIGSITEA^354^	S^40^ D^245^ E^292^ D^319^	H^158^ E^167^ E^210^ E^294^ D^319^ K^344^ R^373^ S^373^ K^395^	^37^SGASTGIH^44^ ^157^CHAGNKLA^164^ ^248^ASEFCKDGKYDLDFKNQTSDPSKH^272^	^251^FCKDGKY^257^	This work
*Bos taurus* (Alpha and beta enolase Q9XSJ4, Q3ZC09; 434 bp)	^340^LLLKVNQIGSTES^353^	S^40^ D^243^ E^289^ D^318^	H^158^ E^167^ E^210^ E^293^ D^318^ K^343^ R^372^ S^373^ K^394^	Alpha enolase ^37^SGASTGIY^44^ ^157^SHAGNKLA^164^ ^248^ASEFYRSGKYDLDFKSPDDPSRYIT^272^ Beta enolase ^248^ASEFYRSGKYDLDFKSPDDPSRYIT^272^	^251^FYRSGKY^257^	This work
*T. cruzi* (KAF8293506.1; 429 bp)	^340^LLLKINQIGTITEA^353^	S^40^ D^243^ E^289^ D^318^	H^156^ E^165^ E^205^ E^291^ D^319^ K^343^ R^372^ S^373^ K^394^	^37^SGASTGIH^44^ ^155^KHAGNALP^162^ ^245^ASETYDENKKQYNLTFKSPEATWVT^270^	^251^FFRSGKY^257^	(56)
*S. pneumoniae* (Q97As2.1; 434 bp)	^340^ILIKVNQIGTLTET^353^	D^242^ E^291^ D^318^	H^155^ E^164^ E^205^ E^291^ D^318^ K^343^ R^372^ S^373^ K^394^	^39^SGASTGEH^46^ ^154^SHSDAPIA^161^ ^245^SSEFYDKERKVYD YTKFEGEGAAVR^269^	^248^FYDKERKVY^256^	(57)
*A. phagocytophilum* (KDB57092.1; 429 bp)	^338^VLIKPNQIGTLSET^351^	S^48^ D^248^ E^289^ D^316^	H^161^ E^170^ E^211^ E^289^ D^316^ K^341^ R^370^ S^371^ K^392^	^45^SGASVGKN^52^ ^160^LHADNGLD^167^ ^250^ASTFYDGKIYKFSG^264^	^254^FYDGKIYK^261^	This work
*A. marginale* MEX-30-184-02 MEX-17-017-01 MEX-30-193-01 MEX-14-010-01 MEX-01-001-01 (RCL19410.1 TZF77690.1 KAB0451331.1 KAB0450913.1 KAB0450361.1; 425 bp)	^338^VLVKPNQIGTLTET^351^	S^48^ D^248^ E^289^ D^316^	H^161^ E^170^ E^211^ E^289^ D^316^ K^341^ R^370^ S^371^ K^392^	^37^SGASVGKF^44^ ^159^ LHADNLLD ^167^ ^250^ASTFYDGKIYKFSG^264^ ^250^ ASTFYDGTSYKFSGK ^264^	^254^FYDGTSYK^261^	This work
*A. marginale* MEX-15-099-01 (KAA8472002.1;450 bp)	^364^VLVKPNQIGTLTET^377^	S^74^ D^274^ E^315^ D^342^	H^187^ E^196^ E^237^ E^291^ D^315^ K^367^ R^396^ S^397^ K^418^	^37^SGASVGKF^44^ ^185^LHADNLLD^193^ ^276^ASTFYDGTSYKFSGK^291^	^37^SGASVGKF^44^ ^165^LHADNLLD^173^ ^256^ASTFYDGTSYKFSGK^271^	This work
*A. marginale* MEX-31-096-01 (KAA8473352.1; 431 bp)	^338^VLIKPNQIGTLSET^351^	S^54^ D^254^ E^295^ D^322^	H^167^ E^176^ E^217^ E^295^ D^322^ K^347^ R^376^ S^377^ K^398^	^279^FYDGTSYK^287^	^259^FYDGTSYK^267^	This work

### 3.4. Three-dimensional (3D) modeling and molecular docking

**Figure 4:**
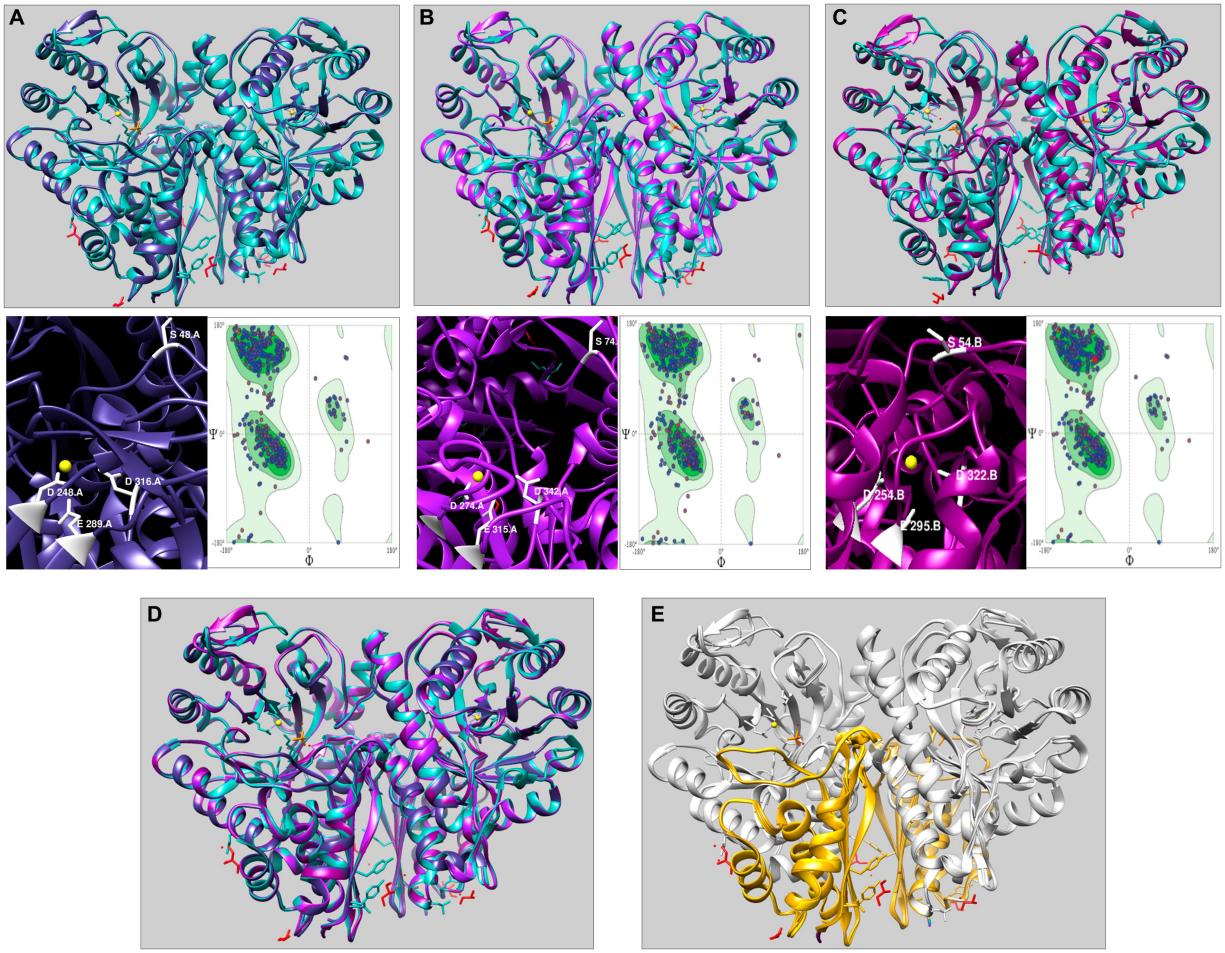
Three-dimensional modeling of enolases from Mexican strains of *A. marginale* using *Enterococcus hirae* enolase as the template (PDB 1IYX, cyan). In the three modeled enolases obtained in Swiss Model **(A)** AmEno01 (purple), **(B)** AmEno15 (magenta), and **(C)** AmEno31, the magnesium binding atoms S, D, E, and D (which vary in position) are shown. Models were considered feasible according to the Ramachandran plots. **(D)** Superposition of AmEno01, AmEno15, AmEno31, and 1IYX. **(E)** Enolases have the topology of a two-layer sandwich in the N-terminal end (golden) and an alpha-beta barrel (TIM barrel) in the C-terminal end (silver). For all models, magnesium atoms are shown in yellow circles; the structure of glycerol molecules is in red, and sulfates in orange. All 3D models were visualized and colored in ChimeraX.

We selected the three representative enolases AmEno01, AmEno15, and AmEno31, as input target sequences in the SwissModel server. The results in SwissModel for templates matching with the target sequence were sorted by higher GMQE value, and the enolase from *Enterococcus hirae* (PDB 1IYX) with a value of GMQE of 0.88 was selected as a template for modeling the three enolases. The identity and coverage percentages between template 1IYX and the modeled AmEno01 were 56.83 and 98%, respectively; for modeled AmEno15, were 56.49 and 92%, respectively; and for modeled AmEno31 were 56.83 and 97%, respectively (Figures 4A–C).

The GMQE value for the modeled enolases AmEno01, AmEno15, and AmEno31 were 0.82, 0.80, and 0.82, respectively, which is a significant accuracy value; hence, these were reliable models. QMEAN value for modeled AmEno01, AmEno15, and AmEno31 was 0.82 ± 0.05.

Since AmEno01, AmEno15, and AmEno31 presented a very similar 3D structure (Figure 4D), with a topology of an alpha-beta barrel (TIM barrel) in the C-terminal end and a two-layer sandwich in the N-terminal end (Figure 4E), we performed the docking with only AmEno01 as a representative model of Mexican strains enolases.

Therefore, the model of AmEno01 was downloaded in PDB format to be used in molecular docking in ClusPro. Five protein–protein dockings were performed in ClusPro, where AmEno01 was considered as a receptor, and the proteins plasminogen (4DUR), fibronectin (3M7P), spectrin (3LBX), ankyrin (4RLV), and stomatin (4FVF), as ligands.

**Figure 5:**
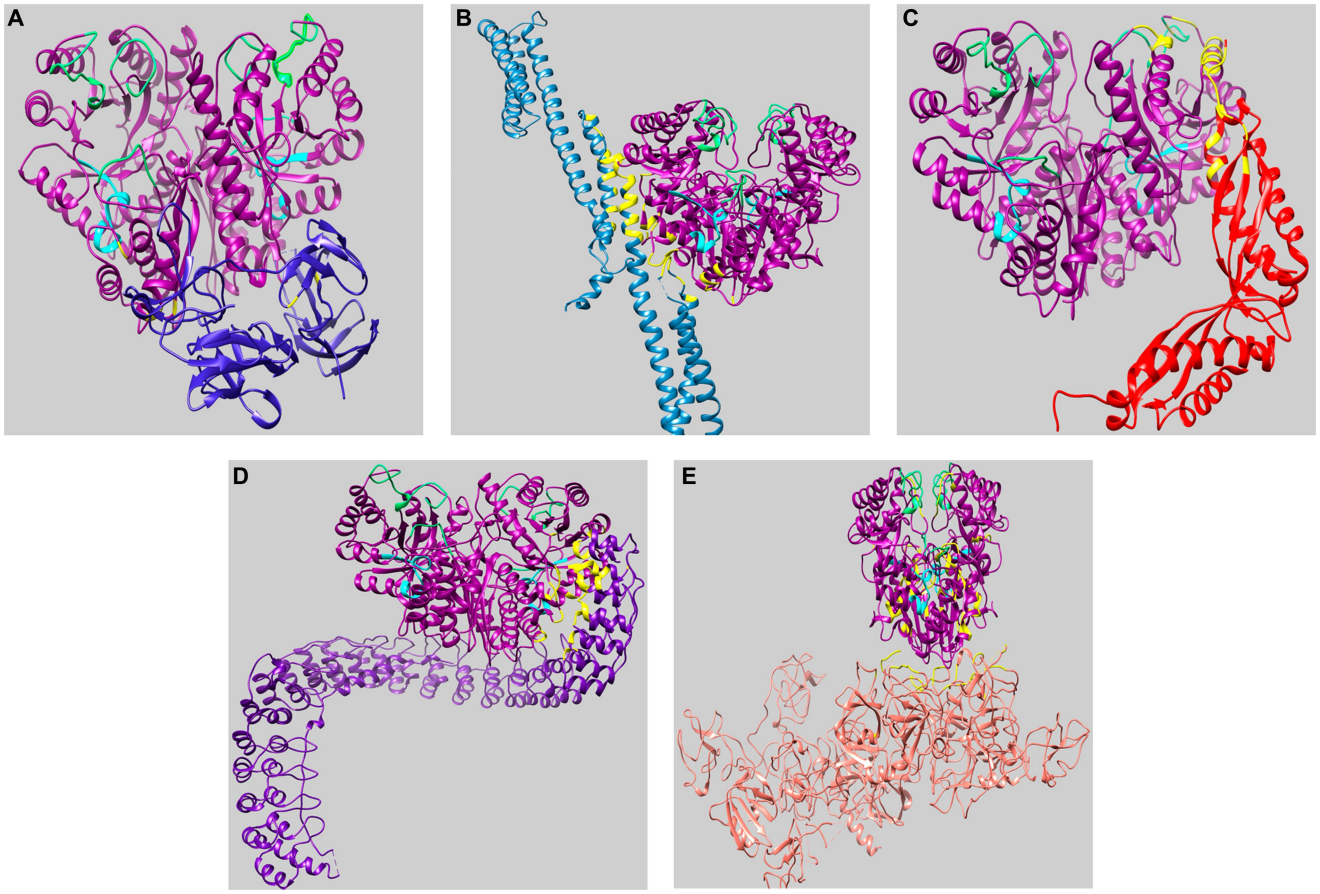
Visualization of molecular docking of AmEno01 with ligands retrieved in ClusPro. **(A)** fibronectin (3M7P); **(B)** spectrin (3LBX); **(C)** stomatin (4FVF); **(D)** ankyrin (4RLV); and **(E)** plasminogen (4DUR). The predicted amino acids participating in the interaction between AmEno01 and each ligand are shown in yellow, the enolase signature is shown in cyan, and the loops of the active site are shown in green. The docked structures were visualized and colored in ChimeraX.

The ten generated docking models by ClusPro were retrieved as “models with balanced coefficients since we do not know what forces dominate the complex protein–protein,” as recommended in the ClusPro manual. One of the ten docking models for each AmEno01-ligand was visualized by UCSF ChimeraX. The molecular dockings are shown in Figure 5, and contact surface models for the five interactions of AmEno01-ligands are shown in Supplementary Figure 2.

In addition, the five molecular dockings were evaluated using the Ramachandran plots to validate the quality of the docking by PDBsum. According to this, the obtained values for each docking were 86.6% (AmEno01-3M7P), 87.3% (AmEno01-3LBX), 86.6% (AmEno01-4FVF), 83.9% (AmEno01-4RLV), and 76.3% (AmEno01-4DUR).

Lastly, the analysis of protein–protein (AmEno01-ligand) performed in PDBsum for the five docking models allowed the identification of the residue interactions across the interface. Thus, the salt bridges, disulfide bonds, hydrogen bonds, and non-bonded contacts were identified for each docking model (Supplementary Figure 3).

## 4. Discussion

The functions reported for enolases as moonlighting proteins in the last years extend their potential applications in the little-explored area of veterinary diseases, such as bovine Anaplasmosis. *A. marginale* is the causal agent of this disease, and seven genomes from Mexican strains have been reported without any moonlighting protein reported in this pathogen up to now (5, 55, 57). However, their presence in genomes of Mexican strains of *A. marginale* suggests they could have similar functions reported elsewhere for this type of protein. The *A. marginale* enolase (AmEno), is of great interest since this protein could have a significant role during the erythrocyte invasion of the pathogen, as it has been reported for *Mycoplasma suis* where the enolase would participate in the adhesion of the pathogen to porcine erythrocytes at early stages of the invasion process (58).

Using bioinformatics approaches allowed us to identify only one enolase sequence per genome in Mexican strains of *A. marginale*, seven enolase sequences. As the phylogenetic reconstruction showed, these sequences grouped with other enolases into three different subclades: Group 1 (five Mexican strains enolases of 425 aa), Group 2 (one Mexican strain enolase of 450 aa and Brazilian strains enolases Palmeira and Jaboticabal), and Group 3 (one Mexican strain enolase of 431 aa and North American strain St. Maries). This suggested that some enolases of Mexican *A. marginale* strains could be related to Brazilian and North American strains, while others could be exclusive to Mexico.

Although the main difference between enolases of Groups 1 to 3 are additional amino acids at the N-terminal end, they have similar secondary structures of beta strands, alpha helix, and coil structures. Additionally, these differences at the sequence level and length observed in Mexican *A. marginale* strains’ enolases did not cause significant modifications in the 3D structural arrangements, as we elucidated when we performed 3D modeling of representative members of each group of enolases: AmEno01, AmEno15, and AmEno31. After predicting 3D structures for these three enolases and realizing their structure was practically identical, we selected AmEno01 for further analysis.

Concerning the AmEno01 3D modeling, our results revealed a dimeric structure of the protein as it has been observed in other enolase structures, including *Helicobacter pylori* (59), *Aeromonas hydrophyla* (60), *Candida albicans* (61), and *Mycoplasma pneumoniae* (62). Additionally, an enolase octameric structure has been reported in *Bacillus subtilis, Streptococcus* spp., and *Thermotoga maritima* (63–65), and a monomeric structure has also been identified *in vitro* conditions (66).

Functionally, in the AmEno01 3D structure, we identified the sequences of the loops of the active site (^37^SGASVGKF^44^, ^159^LHADNLLD^167^, ^250^ASTFYDGKIYKFSG^264^, and ^250^ASTFYDGTSYKFSGK 264) and the enolase signature (^338^VLVKPNQIGTLTET^351^), which were similar to those sequences of the rest of the Mexican enolases’ strains.

On the other hand, it has been reported that two Mg^2+^ ions bind to the amino acids S, D, E, and D in the classical enolase active site, facilitating its catalytic reaction (67). In AmEno01, we found that amino acid S^48^ has a different spatial location than the one observed in other enolases; however we cannot discard its possible binding to the Mg^2+^ ion. In addition, in the predicted 3D models of AmEno01, AmEno15, and AmEno31, the Ramachandran plots, which predict the possible conformation of a protein, revealed that these models with a different spatial location of the S^48^ were feasible.

Subsequently, to analyze the potential of AmEno01 to bind ligands, we explored its interactions with proteins from the extracellular matrix or ECM (fibronectin), erythrocyte membrane (spectrin, ankyrin, and stomatin), and plasminogen by *in silico* approaches.

In this regard, our analysis of the AmEno01-fibronectin interaction showed that the Ramachandran plot contains 86.6% of residues in the most favored regions [A, B, L], suggesting the occurrence possibility of this interaction. The significance of this interaction relies on the ability to bind to fibronectin as a characteristic reported for many pathogens in the early steps of infection of host tissues (68). Interestingly, as *A. marginale* is a pathogen that does not infect tissues, we proposed that the potential of AmEno01 to bind fibronectin is highly relevant to infect ticks if we consider the possibility to adhere to tick gut cells. Like in spirochaete *B. burgdorferi*, whose membrane protein extract interacts with a protein with fibronectin III domains (Ixofin3D) identified in the gut of *Ixodes* spp., facilitating spirochaete congregation to the gut, and providing a molecular exit to the salivary glands before transmission to the human host (69, 70).

On the other hand, we decided to perform a docking analysis to elucidate if AmEno01 could adhere to erythrocyte proteins spectrin and ankyrin as part of the initial invasion process. According to the Ramachandran plot with 87.3 and 83.9% residues, respectively, in the most favored regions, our docking results suggested the binding of AmEno01 with spectrin and ankyrin. Therefore, we hypothesized that AmEno01 adhere to these proteins and probably mediate the initial erythrocyte invasion process. We must highlight that in *A. marginale*, the entry process to the erythrocyte is not well known; however, the ability to attach to host cells is essential for infection (71).

Some examples of enolase as an adhesion molecule to erythrocytes have been reported. Like *M. suis* enolase, that could act as an adhesion factor to porcine erythrocytes. Schreiner et al. (58) found that *M. suis* recombinant enolase bound to erythrocytes lysates in a dose-dependent manner, and even transformants *E. coli* acquired the ability to bind to erythrocytes due to the expression of the enolase on their surface.

In addition to spectrin and ankyrin, another erythrocyte protein is stomatin, an integral protein that plays a role as a membrane-bound scaffolding protein modulating transport protein (72). According to this, the docking results of AmEno01 with stomatin showed 86.6% residues in the most favored regions in the Ramachandran plot. Therefore, we proposed that AmEno01 could recognize stomatin in the erythrocyte membrane as an essential step to adhesion and further internalization. In summary, spectrin, ankyrin, and stomatin could be potential targets to be experimentally assessed to avoid the invasion of *A. marginale* to erythrocytes.

As it is known, *A. marginale* infects erythrocytes but not tissues. Therefore, its binding to plasminogen, a zymogen that facilitates migration and invasion of pathogens to host tissues when converted to plasmin by its proteolytic activity, must be analyzed. Interestingly, we found that 76.3% of residues in the docking AmEno01-plasminogen are in favored regions of the Ramachandran plot, a percentage below the expected values to be considered a good quality model. Additionally, the plasminogen binding sequence identified in Mexican strains of *A. marginale* is different to the sequence of well-known enolases that bind plasminogen (73, 74).

In this regard, we theorized that AmEno01 could be binding to erythrocyte membrane proteins instead of binding to plasminogen; this last one is a strategy that parasites use to invade tissues such as *Leishmania mexicana* (cysticercus), *Taenia solium* and bacteria *S. pneumoniae, Bacillus antracis*, and *B. burgdorferi*, among others (37, 73, 75–77).

Finally, the role of enolases in many organisms is still under study; however, many advances in their functions as moonlighting proteins have been achieved (78). Thus, the study of moonlighting activities attributed to enolases in pathogenic bacteria such as *A. marginale* is relevant since it could be a candidate to control bovine Anaplasmosis. This proposal is based on the approaches performed with moonlighting proteins as potential vaccine candidates against several animal diseases. For example, a robust immune response in mice and piglets was observed when an enolase subunit of *M. suis* was used as antigen (20), or the protective immune response obtained when recombinant enolase of *P. vivax* was expressed in *E. coli* and used as an antigen against malaria (79).

In this regard, elucidating the functions of the enolases from Mexican strains of *A. marginale* could be the basis for developing strategies such as an anti-enolase antibody that interferes, avoids the invasion of erythrocytes, or blocks some other vital processes for the pathogenesis. In addition, we neither exclude that enolases could be proteins driven by the pathogen according to its needs nor discard their possible interaction with tick proteins that contribute to the pathogen’s survival inside the vector.

## Data availability statement

The datasets presented in this study can be found in online repositories. The names of the repository/repositories and accession number(s) can be found in the article/Supplementary Material.

## Author contributions

HA-D and RQ-C conducted the experiments. HA-D, RQ-C, and IA-E analyzed the data. RQ-C and HA-D envisioned and designed the study, and RQ-C and HA-D wrote the manuscript. RQ-C, IA-E, and HA-D edited the manuscript. All authors contributed to the article and approved the submitted version.

## Supplementary material

Supplementary FIGURE 1 **(A)** fibronectin (3M7P); **(B)** spectrin (3LBX); **(C)** stomatin (4FVF) **(D)** ankyrin (4RLV), and **(E)** plasminogen (4DUR).

Supplementary FIGURE 2 **(1)** fibonectin (3M7P); **(2)** spectrin (3LBX); **(3)** stomatin (4FVF) **(4)** ankyrin (4RLV), and **(5)** plasminogen (4DUR).

## References

[1] Krantz M Klipp E. Moonlighting proteins - an approach to systematize the concept. In Silico Biol. (2020) 14:71–83. doi: 10.3233/ISB-190473, PMID: 32285845 PMC7505007

[2] Copley SD. Moonlighting is mainstream: paradigm adjustment required. BioEssays. (2012) 34:578–88. doi: 10.1002/bies.201100191, PMID: 22696112

[3] Arvizu-Rubio VJ García-Carnero LC Mora-Montes HM. Moonlighting proteins in medically relevant fungi. PeerJ. (2022) 10:e14001. doi: 10.7717/peerj.14001, PMID: 36117533 PMC9480056

[4] Satala D Karkowska-Kuleta J Zelazna A Rapala-Kozik M Kozik A. Moonlighting proteins at the candidal cell surface. Microorganisms. (2020) 8:1–25. doi: 10.3390/microorganisms8071046, PMID: 32674422 PMC7409194

[5] Hemmadi V Biswas M. An overview of moonlighting proteins in *Staphylococcus aureus* infection. Arch Microbiol. (2021) 203:481–98. doi: 10.1007/s00203-020-02071-y, PMID: 33048189 PMC7551524

[6] Matos AL Curto P Simões I. Moonlighting in Rickettsiales: expanding virulence landscape. Trop Med Infect Dis. (2022) 7:1–19. doi: 10.3390/tropicalmed7020032 PMC8877226 35202227

[7] Schmid L-M Ohler L Möhlmann T Brachmann A Muiño JM Leister D. PUMPKIN, the sole plastid UMP kinase, associates with group II introns and alters their metabolism. Plant Physiol. (2019) 179:248–64. doi: 10.1104/pp.18.00687 30409856 PMC6324238

[8] Collingridge PW Brown RWB Ginger ML. Moonlighting enzymes in parasitic protozoa. Parasitology. (2010) 137:1467–75. doi: 10.1017/S0031182010000259, PMID: 20233494

[9] Piatigorsky J Wistow GJ. Enzyme/crystallins: gene sharing as an evolutionary strategy. Cells. (1989) 57:197–9. doi: 10.1016/0092-8674(89)90956-2, PMID: 2649248

[10] Wistow G Piatigorsky J. Recruitment of enzymes as lens structural proteins. Science. (1987) 236:1554–6. doi: 10.1126/science.3589669 3589669

[11] Ligabue-Braun R Carlini CR. Moonlighting toxins: ureases and beyond. Plant Toxins. (2015):1–21. doi: 10.1007/978-94-007-6728-7_10-1

[12] Jeffery CJ. Moonlighting proteins: old proteins learning new tricks. Trends Genet. (2003) 19:415–7. doi: 10.1016/S0168-9525(03)00167-7, PMID: 12902157

[13] Jeffery CJ. Protein moonlighting: what is it, and why is itimportant? Phil. Trans. R. Soc. (2017) B373:20160523. doi: 10.1098/rstb.2016.0523

[14] Jeffery CJ. Moonlighting proteins. Trends Biochem Sci. (1999) 24:8–11. doi: 10.1016/s0968-0004(98)01335-8 10087914

[15] Philpott CC Klausner RD Rouault TA. The bifunctional iron-responsive element binding protein/cytosolic aconitase: the role of active-site residues in ligand binding and regulation. Proc Natl Acad Sci U S A. (1994) 91:7321–5. doi: 10.1073/pnas.91.15.7321, PMID: 8041788 PMC44391

[16] Banerjee S Nandyala AK Raviprasad P Ahmed N Hasnain SE. Iron-dependent RNA-binding activity of *Mycobacterium tuberculosis* aconitase. J Bacteriol. (2007) 189:4046–52. doi: 10.1128/JB.00026-07, PMID: 17384188 PMC1913386

[17] Moore B Zhou L Rolland F Hall Q Cheng W-H Liu Y-X. Role of the Arabidopsis glucose sensor HXK1 in nutrient, light, and hormonal signaling. Summarized proceedings for the period from and a directory of members as of. (2003) 300:332–6. doi: 10.1126/science.1080585, PMID: 12690200

[18] Bosch J Buscaglia CA Krumm B Ingason BP Lucas R Roach C. Aldolase provides an unusual binding site for thrombospondin-related anonymous protein in the invasion machinery of the malaria parasite. Proc Natl Acad Sci U S A. (2007) 104:7015–20. doi: 10.1073/pnas.0605301104, PMID: 17426153 PMC1855406

[19] Bedford E Tabor S Richardson CC. The thioredoxin binding domain of bacteriophage T7 DNA polymerase confers processivity on *Escherichia coli* DNA polymerase I. Proc Natl Acad Sci. (1997) 94:479–84. doi: 10.1073/pnas.94.2.479, PMID: 9012809 PMC19538

[20] Xue S Seo K Yang M Cui C Yang M Xiang S. *Mycoplasma suis* alpha-enolase subunit vaccine induces an immune response in experimental animals. Vaccine. (2021) 9:18–20. doi: 10.3390/vaccines9121506, PMID: 34960252 PMC8708218

[21] Hassan M Baig AA Attique SA Abbas S Khan F Zahid S. Molecular docking of alpha-enolase to elucidate the promising candidates against *Streptococcus pneumoniae* infection. Daru. (2021) 29, 73–84., PMID: 33537864 10.1007/s40199-020-00384-3 PMC8149539

[22] Ebner P Götz F. Bacterial excretion of cytoplasmic proteins (ECP): occurrence, mechanism, and function. Trends Microbiol. (2019) 27:176–87. doi: 10.1016/j.tim.2018.10.006, PMID: 30442534

[23] Wang G Xia Y Cui J Gu Z Song Y Chen YQ. The roles of moonlighting proteins in Bacteria. Curr Issues Mol Biol. (2014) 16:15–22. doi: 10.21775/cimb.016.015 23872606

[24] Franco-Serrano L Sánchez-Redondo D Nájar-García A Hernández S Amela I Perez-Pons JA. Pathogen moonlighting proteins: from ancestral key metabolic enzymes to virulence factors. Microorganisms. (2021) 9:1300. doi: 10.3390/microorganisms9061300, PMID: 34203698 PMC8232316

[25] Ko K-C Lee JH Han Y Choi JH Song JJ. A novel multifunctional cellulolytic enzyme screened from metagenomic resources representing ruminal bacteria. Biochem Biophys Res Commun. (2013) 441:567–72. doi: 10.1016/j.bbrc.2013.10.120, PMID: 24184482

[26] Jeffery CJ. Intracellular/surface moonlighting proteins that aid in the attachment of gut microbiota to the host. AIMS Microbiol. (2019) 5:77–86. doi: 10.3934/microbiol.2019.1.77, PMID: 31384704 PMC6646928

[27] Commichau FM Stülke J. Trigger enzymes: coordination of metabolism and virulence gene expression. Microbiol Spectr. (2015) 3:105–127. doi: 10.1128/microbiolspec.MBP-0010-2014, PMID: 26350309

[28] Kainulainen V Korhonen TK. Dancing to another tune—adhesive moonlighting proteins in Bacteria. Biology (Basel). (2014) 3:178–204. doi: 10.3390/biology3010178, PMID: 24833341 PMC4009768

[29] Theiler A. Further investigation into anaplasmosis of south African cattle In: Theiler A, editor. First report of the director of veterinary research. Department of Agriculture of the Union of South Africa: South Africa (1911). 7–47.

[30] Aubry P Geale DW. A review of bovine anaplasmosis. Transbound Emerg Dis. (2011) 58:1–30. doi: 10.1111/j.1865-1682.2010.01173.x 21040509

[31] Quiroz-Castañeda RE Amaro Estrada I Martínez Ocampo F Rodríguez Camarillo S Dantán González E Cobaxin Cárdenas M. Draft genome sequence of *Anaplasma marginale* strain MEX-01-001-01, a mexican strain that causes bovine anaplasmosis. Microbiol Resour Announc. (2018) 7:e01101–18. doi: 10.1128/MRA.01101-18, PMID: 30533750 PMC6256586

[32] Martínez-Ocampo F Quiroz-Castañeda RE Amaro-Estrada I Cobaxin Cárdenas M Dantán-González E Rodríguez-Camarillo S. Draft genome sequences of *Anaplasma marginale* strains MEX-15-099-01 and MEX-31-096-01, two Mexican isolates with different degrees of virulence. Microbiol Resour Announc. (2019) 8. doi: 10.1128/MRA.01184-19, PMID: 31699769 PMC6838627

[33] Martínez-Ocampo F Quiroz-Castañeda RE Amaro-Estrada I Dantán-González E De La Torre JFP Rodríguez-Camarillo S. Whole-genome sequencing of Mexican strains of *Anaplasma marginale*: an approach to the causal agent of bovine Anaplasmosis. Int J Genomics. (2020) 2020:1–7. doi: 10.1155/2020/5902029, PMID: 32351981 PMC7178543

[34] Rodríguez-Camarillo SD Quiroz-Castañeda RE Aguilar-Díaz H Vara-Pastrana JE Pescador-Pérez D Amaro-Estrada I. Immunoinformatic analysis to identify proteins to be used as potential targets to control bovine Anaplasmosis. Int J Microbiol. (2020) 2020:8882031–8. doi: 10.1155/2020/8882031, PMID: 32908531 PMC7474394

[35] Gao X Zheng C Liu X. Expression, purification, and biological characterization of *Anaplasma phagocytophilum* enolase. Biosci Trends. (2018) 11:651–7. doi: 10.5582/bst.2017.01195, PMID: 29249727

[36] Nogueira SV Smith AA Qin J-H Pal U. A surface enolase participates in *Borrelia burgdorferi*-plasminogen interaction and contributes to pathogen survival within feeding ticks. Infect Immun. (2012) 80:82–90. doi: 10.1128/IAI.05671-11, PMID: 22025510 PMC3255677

[37] Floden AM Watt JA Brissette CA. *Borrelia burgdorferi* enolase is a surface-exposed plasminogen binding protein. PLoS One. (2011) 6:e27502. doi: 10.1371/journal.pone.0027502, PMID: 22087329 PMC3210797

[38] Xie Q Xing H Wen X Liu B Wei Y Yu Y. Identification of the multiple roles of enolase as an plasminogen receptor and adhesin in *Mycoplasma hyopneumoniae*. Microb Pathog. (2023) 174:105934. doi: 10.1016/j.micpath.2022.105934, PMID: 36481292

[39] Aziz RK Bartels D Best AA DeJongh M Disz T Edwards RA. The RAST server: rapid annotations using subsystems technology. BMC Genomics. (2008) 9:75. doi: 10.1186/1471-2164-9-75, PMID: 18261238 PMC2265698

[40] Jumper J Evans R Pritzel A Green T Figurnov M Ronneberger O. Highly accurate protein structure prediction with AlphaFold. Nature. (2021) 596:583–9. doi: 10.1038/s41586-021-03819-2, PMID: 34265844 PMC8371605

[41] Chen C Zabad S Liu H Wang W Jeffery C. MoonProt 2.0: an expansion and update of the moonlighting proteins database. Nucleic Acids Res. (2018) 46:D640–4. doi: 10.1093/nar/gkx1043, PMID: 29126295 PMC5753272

[42] Sievers F Wilm A Dineen D Gibson TJ Karplus K Li W. Fast, scalable generation of high-quality protein multiple sequence alignments using Clustal omega. Mol Syst Biol. (2011) 7:539. doi: 10.1038/msb.2011.75, PMID: 21988835 PMC3261699

[43] Waterhouse AM Procter JB Martin DMA Clamp M Barton GJ. Jalview version 2--a multiple sequence alignment editor and analysis workbench. Bioinformatics. (2009) 25:1189–91. doi: 10.1093/bioinformatics/btp033, PMID: 19151095 PMC2672624

[44] Tamura K Stecher G Kumar S. MEGA11: molecular evolutionary genetics analysis version 11. Mol Biol Evol. (2021) 38:3022–7. doi: 10.1093/molbev/msab120, PMID: 33892491 PMC8233496

[45] Sigrist CJA de Castro E Cerutti L Cuche BA Hulo N Bridge A. New and continuing developments at PROSITE. Nucleic Acids Res. (2013) 41:D344–7. doi: 10.1093/nar/gks1067, PMID: 23161676 PMC3531220

[46] Yu NY Wagner JR Laird MR Melli G Rey S Lo R. PSORTb 3.0: improved protein subcellular localization prediction with refined localization subcategories and predictive capabilities for all prokaryotes. Bioinformatics. (2010) 26:1608–15. doi: 10.1093/bioinformatics/btq249, PMID: 20472543 PMC2887053

[47] Buchan DWA Jones DT. The PSIPRED protein analysis workbench: 20 years on. Nucleic Acids Res. (2019) 47:W402–7. doi: 10.1093/nar/gkz297, PMID: 31251384 PMC6602445

[48] Sillitoe I Bordin N Dawson N Waman VP Ashford P Scholes HM. CATH: increased structural coverage of functional space. Nucleic Acids Res. (2020) 49:D266–73. doi: 10.1093/nar/gkaa1079, PMID: 33237325 PMC7778904

[49] Waterhouse A Bertoni M Bienert S Studer G Tauriello G Gumienny R. SWISS-MODEL: homology modelling of protein structures and complexes. Nucleic Acids Res. (2018) 46:W296–303. doi: 10.1093/nar/gky427, PMID: 29788355 PMC6030848

[50] Kozakov D Hall DR Xia B Porter KA Padhorny D Yueh C. The ClusPro web server for protein–protein docking. Nat Protoc. (2017) 12:255–78. doi: 10.1038/nprot.2016.169, PMID: 28079879 PMC5540229

[51] Desta IT Porter KA Xia B Kozakov D Vajda S. Performance and its limits in rigid body protein-protein docking. Structure. (2020) 28:1071–1081.e3. doi: 10.1016/j.str.2020.06.006, PMID: 32649857 PMC7484347

[52] Goddard TD Huang CC Meng EC Pettersen EF Couch GS Morris JH. UCSF ChimeraX: meeting modern challenges in visualization and analysis. Protein Sci. (2018) 27:14–25. doi: 10.1002/pro.3235, PMID: 28710774 PMC5734306

[53] Yan Y Tao H He J Huang S-Y. The HDOCK server for integrated protein–protein docking. Nat Protoc. (2020) 15:1829–52. doi: 10.1038/s41596-020-0312-x, PMID: 32269383

[54] Laskowski RA Jabłońska J Pravda L Vařeková RS Thornton JM. PDBsum: structural summaries of PDB entries. Protein Sci. (2018) 27:129–34. doi: 10.1002/pro.3289, PMID: 28875543 PMC5734310

[55] Kang HJ Jung S-K Kim SJ Chung SJ. Structure of human {$α$}-enolase (hENO1), a multifunctional glycolytic enzyme. Acta Crystallogr Sect D. (2008) 64:651–7. doi: 10.1107/S0907444908008561, PMID: 18560153

[56] Diaz-Hernandez A Gonzalez-Vazquez MC Arce-Fonseca M Rodríguez-Morales O Cedillo-Ramirez ML Carabarin-Lima A. Consensus enolase of Trypanosoma Cruzi: Evaluation of their immunogenic properties using a bioinformatics approach. Life (Basel, Switzerland). (2022) 12:12050746. doi: 10.3390/life12050746, PMID: 35629412 PMC9148029

[57] Ehinger S Schubert W-D Bergmann S Hammerschmidt S Heinz DW. Plasmin(ogen)-binding α-enolase from *Streptococcus pneumoniae*: crystal structure and evaluation of plasmin(ogen)-binding sites. J Mol Biol. (2004) 343:997–1005. doi: 10.1016/j.jmb.2004.08.088 15476816

[58] Schreiner SA Sokoli A Felder KM Wittenbrink MM Schwarzenbach S Guhl B. The surface-localised α-enolase of *Mycoplasma suis* is an adhesion protein. Vet Microbiol. (2012) 156:88–95. doi: 10.1016/j.vetmic.2011.10.010, PMID: 22047714

[59] López-López MJ Rodríguez-Luna IC Lara-Ramírez EE López-Hidalgo M Benítez-Cardoza CG Guo X. Biochemical and biophysical characterization of the enolase from *Helicobacter pylori*. Biomed Res Int. (2018) 2018:9538193. doi: 10.1155/2018/9538193, PMID: 30648111 PMC6311853

[60] Sha J Erova TE Alyea RA Wang S Olano JP Pancholi V. Surface-expressed enolase contributes to the pathogenesis of clinical isolate SSU of *Aeromonas hydrophila*. J Bacteriol. (2009) 191:3095–107. doi: 10.1128/JB.00005-09, PMID: 19270100 PMC2681796

[61] Satala D Satala G Karkowska-Kuleta J Bukowski M Kluza A Rapala-Kozik M. Structural insights into the interactions of Candidal enolase with human Vitronectin, fibronectin and plasminogen. Int J Mol Sci. (2020) 21. doi: 10.3390/ijms21217843, PMID: 33105833 PMC7660097

[62] Chen R Zhao L Gan R Feng Z Cui C Xie X. Evidence for the rapid and divergent evolution of mycoplasmas: structural and phylogenetic analysis of enolases. Front Mol Biosci. (2022) 8:811106. doi: 10.3389/fmolb.2021.811106, PMID: 35145997 PMC8822174

[63] Newman JA Hewitt L Rodrigues C Solovyova AS Harwood CR Lewis RJ. Dissection of the network of interactions that links RNA processing with glycolysis in the *Bacillus subtilis* Degradosome. J Mol Biol. (2012) 416:121–36. doi: 10.1016/j.jmb.2011.12.024, PMID: 22198292

[64] Lu Q Lu H Qi J Lu G Gao GF. An octamer of enolase from *Streptococcus suis*. Protein Cell. (2012) 3:769–80. doi: 10.1007/s13238-012-2040-7, PMID: 23055041 PMC4875344

[65] Schurig H Rutkat K Jaenicke R Rachel R. Octameric enolase from the hyperthermophilic bacterium *Thermotoga maritima*: purification, characterization, and image processing. Protein Sci. (1995) 4:228–36. doi: 10.1002/pro.5560040209, PMID: 7757011 PMC2143061

[66] Mirasol-Meléndez E Lima E Lara V Brieba LG Lara-González S Benitez-Cardoza CG. Self-Association of Enolase from trichomonas vaginalis. Monomers, dimers, and octamers coexist in solution. ACS. Omega. (2018) 3:17871–80. doi: 10.1021/acsomega.8b02197

[67] Schreier B Höcker B. Engineering the enolase magnesium II binding site: implications for its evolution. Biochemistry. (2010) 49:7582–9. doi: 10.1021/bi100954f, PMID: 20690637

[68] Joh D Wann ER Kreikemeyer B Speziale P Höök M. Role of fibronectin-binding MSCRAMMs in bacterial adherence and entry into mammalian cells. Matrix Biol. (1999) 18:211–23. doi: 10.1016/s0945-053x(99)00025-6, PMID: 10429941

[69] Narasimhan S Coumou J Schuijt TJ Boder E Hovius JW Fikrig E. A tick gut protein with fibronectin III domains aids *Borrelia burgdorferi* congregation to the gut during transmission. PLoS Pathog. (2014) 10:e1004278–8. doi: 10.1371/journal.ppat.1004278, PMID: 25102051 PMC4125277

[70] Kurokawa C Lynn GE Pedra JHF Pal U Narasimhan S Fikrig E. Interactions between Borrelia burgdorferi and ticks. Nat Rev Microbiol. (2020) 18:587–600. doi: 10.1038/s41579-020-0400-5, PMID: 32651470 PMC7351536

[71] Rikihisa Y. Mechanisms of obligatory intracellular infection with *Anaplasma phagocytophilum*. Clin Microbiol Rev. (2011) 24:469–89. doi: 10.1128/CMR.00064-10, PMID: 21734244 PMC3131063

[72] Genetet S Desrames A Chouali Y Ripoche P Lopez C Mouro-Chanteloup I. Stomatin modulates the activity of the anion exchanger 1 (AE1, SLC4A1). Sci Rep. (2017) 7:46170. doi: 10.1038/srep46170, PMID: 28387307 PMC5383999

[73] Vanegas G Quiñones W Carrasco-López C Concepción JL Albericio F Avilán L. Enolase as a plasminogen binding protein in Leishmania mexicana. Parasitol Res. (2007) 101:1511–6. doi: 10.1007/s00436-007-0668-7, PMID: 17653767

[74] Pancholi V Fischetti VA. Α-enolase, a novel strong plasmin(Ogen) binding protein on the surface of pathogenic streptococci. J Biol Chem. (1998) 273:14503–15. doi: 10.1074/jbc.273.23.14503, PMID: 9603964

[75] Ayón-Núñez DA Fragoso G Espitia C García-Varela M Soberón X Rosas G. Identification and characterization of Taenia solium enolase as a plasminogen-binding protein. Acta Trop. (2018) 182:69–79. doi: 10.1016/j.actatropica.2018.02.020, PMID: 29466706

[76] Bergmann S Schoenen H Hammerschmidt S. The interaction between bacterial enolase and plasminogen promotes adherence of *Streptococcus pneumoniae* to epithelial and endothelial cells. Int J Med Microbiol. (2013) 303:452–62. doi: 10.1016/j.ijmm.2013.06.002, PMID: 23906818

[77] Agarwal S Kulshreshtha P Bambah Mukku D Bhatnagar R. Alpha-enolase binds to human plasminogen on the surface of *Bacillus anthracis*. Biochim Biophys Acta. (2008) 1784:986–94. doi: 10.1016/j.bbapap.2008.03.017, PMID: 18456007

[78] Didiasova M Schaefer L Wygrecka M. When place matters: shuttling of enolase-1 across cellular compartments. Front cell. Dev Biol. (2019) 7:61. doi: 10.3389/fcell.2019.00061, PMID: 31106201 PMC6498095

[79] Zhang C Gu Y Tang J Lu F Cao Y Zhou H. Production of plasmodium vivax enolase in Escherichia coli and its protective properties. Hum Vaccin Immunother. (2016) 12:2855–61. doi: 10.1080/21645515.2016.1208328, PMID: 27487171 PMC5137521

