# Supplementary figures and images for: An alternative vaccine target for bovine Anaplasmosis based on enolase, a moonlighting protein
Source: Front Vet Sci. 2023 Sep 22;10:1225873. doi: 10.3389/fvets.2023.1225873 (PMC10556744; doi:10.3389/fvets.2023.1225873)

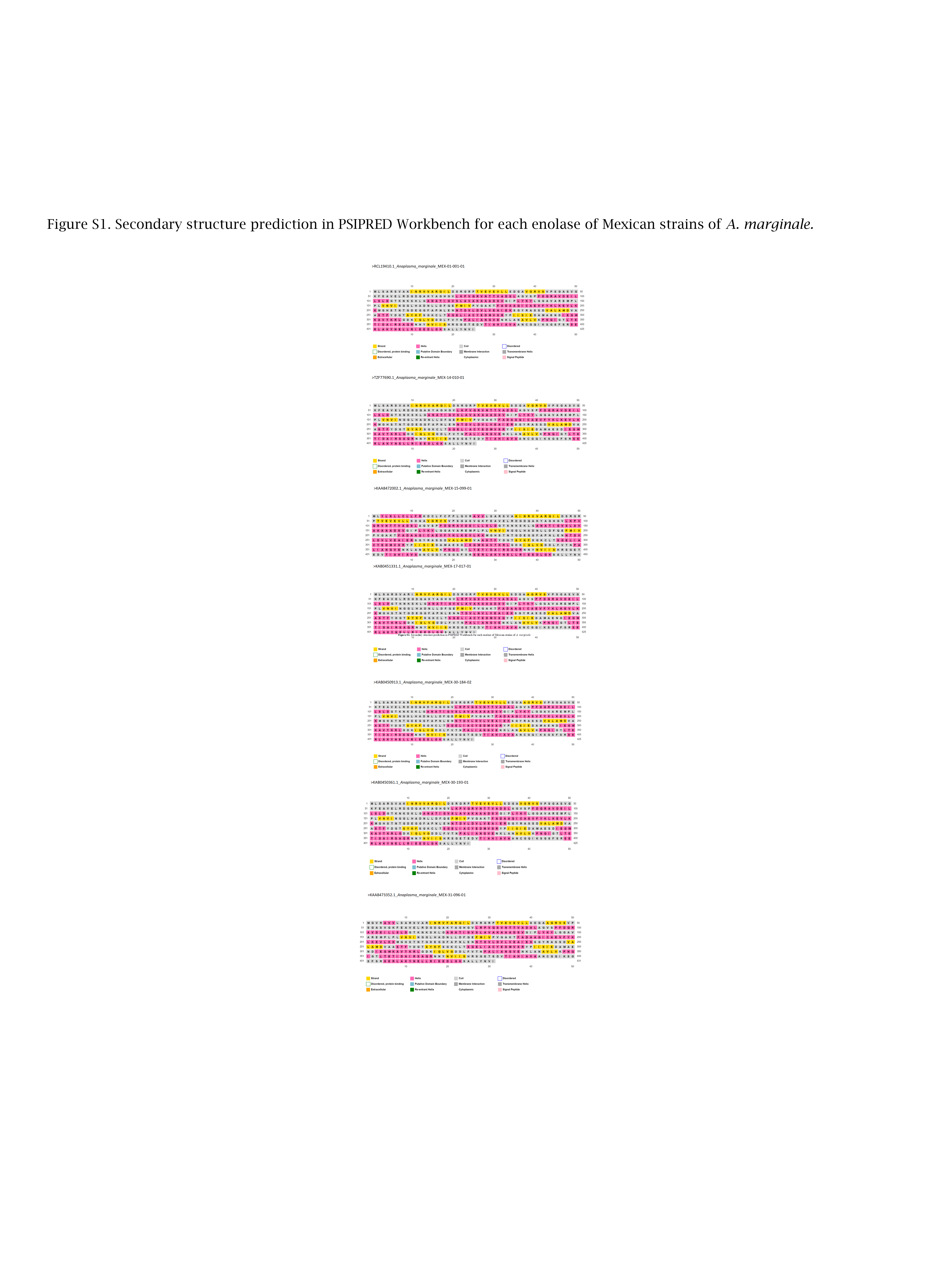

Supplement: Supplementary FIGURE 1 — (A) fibronectin (3M7P); (B) spectrin (3LBX); (C) stomatin (4FVF) (D) ankyrin (4RLV), and (E) plasminogen (4DUR). [file Image_1.TIF]

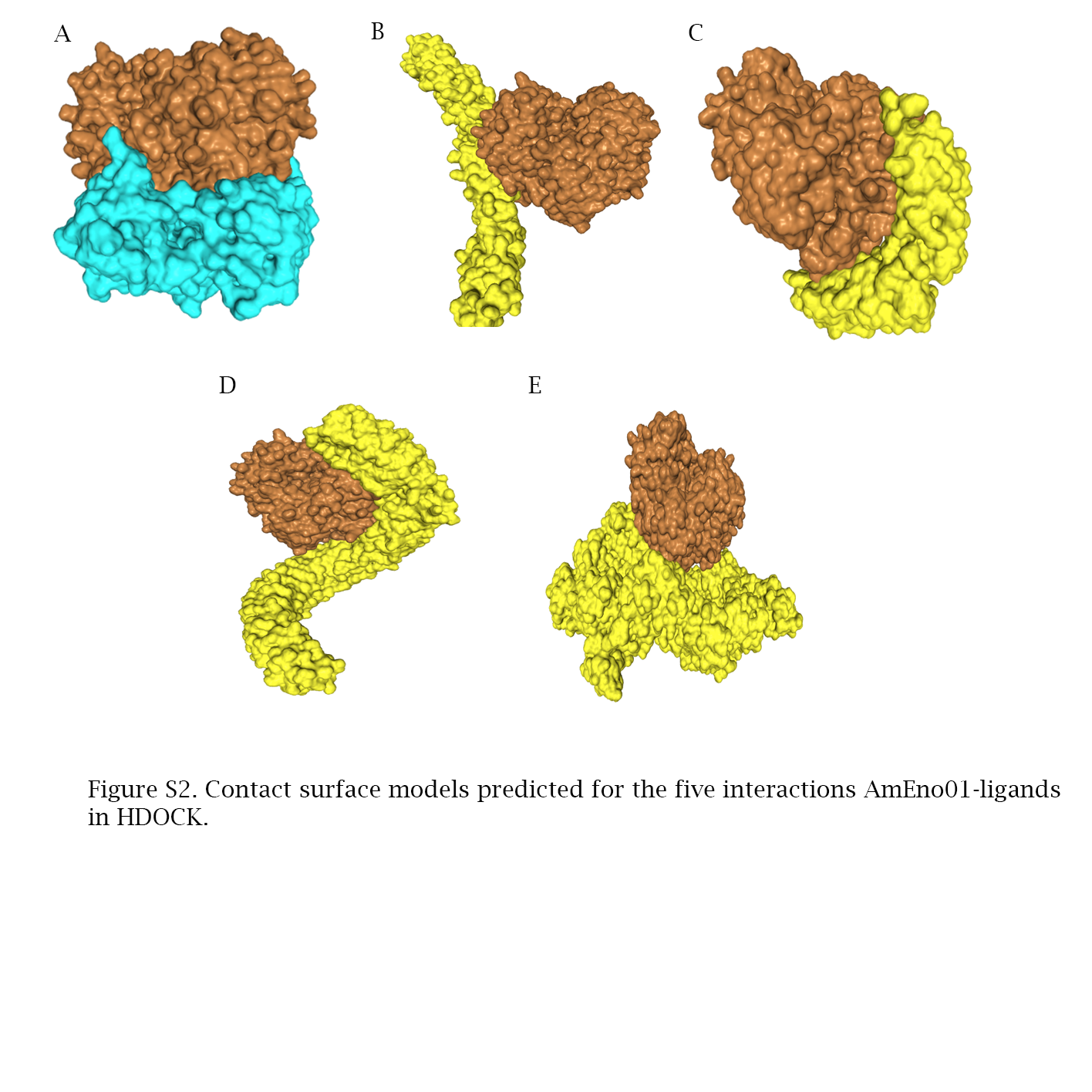

Supplement: Supplementary FIGURE 2 — (1) fibonectin (3M7P); (2) spectrin (3LBX); (3) stomatin (4FVF) (4) ankyrin (4RLV), and (5) plasminogen (4DUR). [file Image_2.TIF]

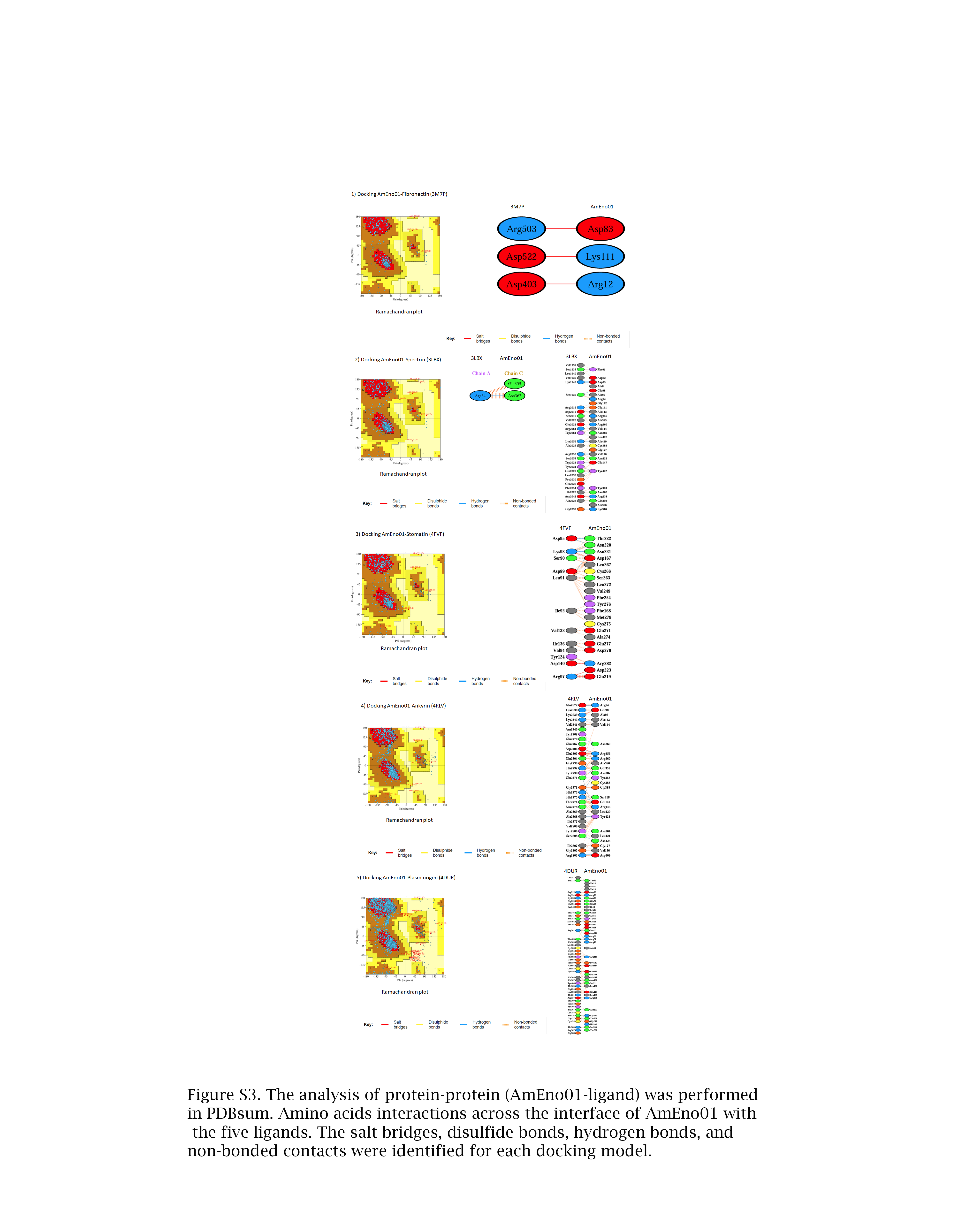

Supplement: Supplementary file 3 [file Image_3.TIF]
