# Supplementary material for: An alternative vaccine target for bovine Anaplasmosis based on enolase, a moonlighting protein
Source: Front Vet Sci. 2023 Sep 22;10:1225873. doi: 10.3389/fvets.2023.1225873 (PMC10556744; doi:10.3389/fvets.2023.1225873)
Supplement: Supplementary file 4 [file Table_1.pdf]

Table S1. Accesion numbers of the enolases from Domains Eukarya and Bacteria used in this work.

**Domain**	
**Eukarya**	**Animalia** (P04764, P17182, Q9XSJ4, XP_020950937.1, P06733, P07323, P09104, P17183, P13929, Q1KYT0, Q3ZC09, P15429, CAA40913.1, P21550);<br>**Protist** (ESU44637.1, KAF8293506.1);<br>**Arthropoda** (XP_029835840.2, JAP86447.1, QTX16297).
**Bacteria**	**Bacteria** (AZV75045.1, PLQ64569.1, WP06543266.1, WP151807858.1, KDB57092.1, AGR81391.1, AXW85477.1, AXW84543.1, KAA8472002.1, KAB0450361.1, TZF77690.1, RCL19410.1, KAB0450913.1, KAB0451331.1, KAA8473352.1, AAV86604.1, WP010889730.1).
